# A fast and fully-automated deep-learning approach for accurate hemorrhage segmentation and volume quantification in non-contrast whole-head CT

**DOI:** 10.1038/s41598-020-76459-7

**Published:** 2020-11-09

**Authors:** Ali Arab, Betty Chinda, George Medvedev, William Siu, Hui Guo, Tao Gu, Sylvain Moreno, Ghassan Hamarneh, Martin Ester, Xiaowei Song

**Affiliations:** 1 School of Computing Science, Simon Fraser University, Burnaby, BC Canada; 2 Department of Biomedical Physiology and Kinesiology, Simon Fraser University, Burnaby, BC Canada; 3 Health Sciences and Innovation, Surrey Memorial Hospital, Fraser Health Authority, Surrey, BC Canada; 4 Division of Neurology, Royal Columbian Hospital, New Westminster, BC Canada; 5 Division of Radiology, Royal Columbian Hospital, New Westminster, BC Canada; 6 Department of Radiology, Tianjin Medical University General Hospital, Tianjin, China; 7 Department of Radiology, Beijing Hospital, Beijing, China; 8 School of Interactive Arts and Technology, Simon Fraser University, Surrey, BC Canada; 9 Digital Health Circle, Simon Fraser University, Surrey, BC Canada

**Keywords:** Stroke, Brain imaging, Machine learning, Image processing, Computational models

## Abstract

This project aimed to develop and evaluate a fast and fully-automated deep-learning method applying convolutional neural networks with deep supervision (CNN-DS) for accurate hematoma segmentation and volume quantification in computed tomography (CT) scans. Non-contrast whole-head CT scans of 55 patients with hemorrhagic stroke were used. Individual scans were standardized to 64 axial slices of 128 × 128 voxels. Each voxel was annotated independently by experienced raters, generating a binary label of hematoma versus normal brain tissue based on majority voting. The dataset was split randomly into training (n = 45) and testing (n = 10) subsets. A CNN-DS model was built applying the training data and examined using the testing data. Performance of the CNN-DS solution was compared with three previously established methods. The CNN-DS achieved a Dice coefficient score of 0.84 ± 0.06 and recall of 0.83 ± 0.07, higher than patch-wise U-Net (< 0.76). CNN-DS average running time of 0.74 ± 0.07 s was faster than PItcHPERFeCT (> 1412 s) and slice-based U-Net (> 12 s). Comparable interrater agreement rates were observed between “method-human” vs. “human–human” (Cohen’s kappa coefficients > 0.82). The fully automated CNN-DS approach demonstrated expert-level accuracy in fast segmentation and quantification of hematoma, substantially improving over previous methods. Further research is warranted to test the CNN-DS solution as a software tool in clinical settings for effective stroke management.

## Introduction

Hemorrhagic stroke refers to the loss of brain function due to the accumulation of blood inside the brain arising from compromised cerebral vasculature^1,2^. It is associated with high death rate and low recovery probability^3,4^. Over 60 million DALY’s (Disability Adjusted Life Years—a measure of lost time and economic resources) are lost annually to hemorrhagic stroke^5^, with stroke care costing 34 billion dollars in the United States alone^6^.

Based on the stroke best practice recommendations, the presence of acute hemorrhagic stroke is confirmed clinically using non-contrast computed tomography (CT) imaging, which visibly distinguishes pathologic blood from normal brain tissue^7^. Precision of clinical decision making for hemorrhagic stroke is greatly enhanced by the *timely* and *accurate* retrieval and application of information embedded within these scans. Hematoma detection and assessment of the volume, location, and spread of the bleed are contingent for correct prognostication and treatment outcomes, constituting major determinants of stroke mortality^8–11^.

However, the paucity of fast and accurate hematoma volumetric analysis tools have limited the effective utility of CT-based information in guidance of care plans. Current clinical practice for hematoma quantification employs primarily the simple ABC (length, width, and height)/2 method, which assumes that the hemorrhage is elliptical and accordingly estimates hemorrhage volume by calculating the volume of the ellipse^12^. Research even till recent has consistently shown that manual calculations involved with this clinical standard can be inherently time-consuming, expertise dependent, error-prone, and challenging to deal with several complex, large, and irregular hematoma cases^11^.

Given the clinical significance, numerous attempts at developing computer-assisted automation tools for hematoma volume have been reported^13–16^. However, many of the techniques even till recent are either manual, semi-automatic, slow, or suffer from poor accuracy, especially for irregular hematoma shapes and sizes^13–19^. Great efforts have been geared towards building automated segmentation tools with use of artificial intelligence technologies, in particular the promising deep-learning algorithms^20,21^. Even so, established methods thus far have been largely restricted to handling intracranial hemorrhage and have not been evaluated in clinical settings^20,21^. Developing fully-automated deep-learning methods with both high-level of accuracy and efficiency for improved clinical management of multiple types of hemorrhagic stroke is holding tremendous research attention^22,23^.

Previously proposed approaches have attempted to address the problem through optimizing a hematoma mask using level-set techniques for computations of the hematoma surfaces and shapes^24–27^. Despite the ease to follow complex shapes without the prerequisite of complicated parameterization, the level-set based methods have generally suffered from oversensitivity to the contour initialization, susceptibility to local optima and requiring numerous convergence iterations^24–27^.

Alternatively, recent research has applied supervised learning methods, where the algorithm can learn the hematoma mapping function under supervision from the ground truth, i.e., previously defined labels indicating the hematoma regions. For example, the well-established PItcHPERFeCT method computes hand-engineered features (i.e., extract useful information from the images using the domain knowledge) for each voxel based on the surrounding patch, and a random forest model is trained to classify each voxel^28^. However, despite the high accuracy, PItcHPERFeCT is slow as its feature extraction is computationally very expensive^28^.

As an important aspect of artificial intelligence, the deep learning approaches have drawn an increasingly great attention in medical imaging analyses, due to its abilities to learn non-linear relationships from the data and to perform automatic feature extraction without using any domain knowledge. Several deep learning applications have tackled intracerebral hemorrhage (ICH) segmentation^17,29,30^. Many of the methods are based on the U-Net model, a deep neural network based approach designed for training end-to-end for dense segmentation, i.e., producing voxel-wise labelling^7,29^. Although the currently available deep learning methods have shown effectiveness and efficiency in segmenting ICH, there are unsolved questions calling for further research. First, most of the previous findings were based on CT images from a single institute and thus requires generalization testing. The model's performance might be affected by slice spacing that can vary between CT scans in multiple center data. Also, the input data to most of the methods were only patches or a small number of consecutive slices of the CT scan, which limited the model's field of view while requiring additional inferences to reconstruct the whole-brain images, increasing the time for the error-prone prediction.

Here, our team aimed to develop a fast and fully-automated deep-learning oriented approach for accurate hemorrhage segmentation and volume quantification on non-contrast whole-head CT^31^. To achieve this goal, we applied a convolutional neural network with deep supervision (CNN-DS) on a novel dataset collected from three hospitals. This modified U-Net with deep supervision method combined the strengths of CNN in effectively learning visual imagery data with deep supervision to speed up the convergence and reduce over-fitting^32^, and thereby increasing generalizability of the model. This ability is crucial in studying medical images where training sample size is often limited by the availability of clinical data and human-expert labeling^32^. This study marks the first effort to our knowledge that extends the usability of U-Net with deep supervision for the segmentation and quantification of hematoma volume in CT scans. We evaluated the performance of the CNN-DS method in terms of accuracy and efficiency, and compared these with those of three recently established well-adopted machine-learning algorithms. We hypothesized that the CNN-DS approach can yield an expert-level of accuracy in segmenting and evaluating hemorrhage volume on head CT images, while being highly time-efficient.

## Results

### Training and test cohort

Table 1 provides the characteristics of the patients randomly selected for the training and testing data subsets. Patients in the two subsets showed similar demographics, even though patients in the testing set appeared to be slightly older (t = − 1.6, p = 0.12 two-tailed).

**Table 1:** Patient demographics.

	Training set	Testing set
N	45	10
Age (years, mean ± std)	62.6 ± 15.7	70.7 ± 8.4
Sex (male, %)	51	60
Deep-seated intracerebral hemorrhage (ICH, %)	40	30
Hemorrhage reaching cortical surface (%)	77.8	100
Intraventricular hemorrhage (%)	40	30
Oral anticoagulants (%)	24.4	40
Antiplatelet (%)	6.7	0
Hematoma evacuation (%)	11.1	0

### Segmentation accuracy, efficiency, and reliability

The CNN-DS method for hematoma quantification in the training set had a Dice coefficient score of 0.82 ± 0.06 at an average running time of 0.59 ± 0.02 s.

Compared with the reference standard of expert labelling, the CNN-DS method resulted in a Dice similarity coefficient, precision, and recall of 0.84 ± 0.06, 0.85 ± 0.07 and 0.83 ± 0.07 respectively in the testing set. The Cohen’s kappa coefficient for each pair of raters was consistently high as shown in Table 2 (M indicates the CNN-DS method; ${E}_{i},$ human-expert rater *i*; CI, 95% confidence interval; kappa, Cohen’s kappa coefficient; β and α, slopes and intercepts in the regressed linear model; * indicates p < 0.001.). The average of the kappa coefficient for inter-expert pairs in {E1, E2, E3} was 0.88 ± 0.01 and the corresponding value for “method-expert” pairs was 0.83 ± 0.01. For the two groups containing kappa coefficient measures for all images and all raters, there was no statistically significant difference in the mean between “expert-expert” and “method-expert” pairs (Mann Whitney U, p > 0.05). In addition, the estimated parameters of linear regression showed significant (p_β_ ≤ 0.002) correlations and insignificant intercept (p_α_ ≥ 0.09) between different pairs (Table 2).

**Table 2:** Agreement measures for each pair of raters on the testing data set.

Raters	Cohen’s kappa	β (p-value)	α (p-value)
E1, E2	0.88 ± 0.05	1.13 (p = 7.99e−10*)	− 2.30 (p = 0.141)
E1, E3	0.87 ± 0.07	1.45 (p = 1.11e−06*)	− 8.26 (p = 0.107)
E2, E3	0.89 ± 0.07	1.29 (p = 1.15e−07*)	− 5.60 (p = 0.126)
E1, M	0.84 ± 0.06	1.05 (p = 6.12e−08*)	− 1.24(p = 0.600)
E2, M	0.82 ± 0.08	0.93 (p = 4.97e−08*)	1.01 (p = 0.648)
E3, M	0.82 ± 0.08	0.69 (p = 1.31e−05*)	6.36 (p = 0.142)

**Figure 1:**
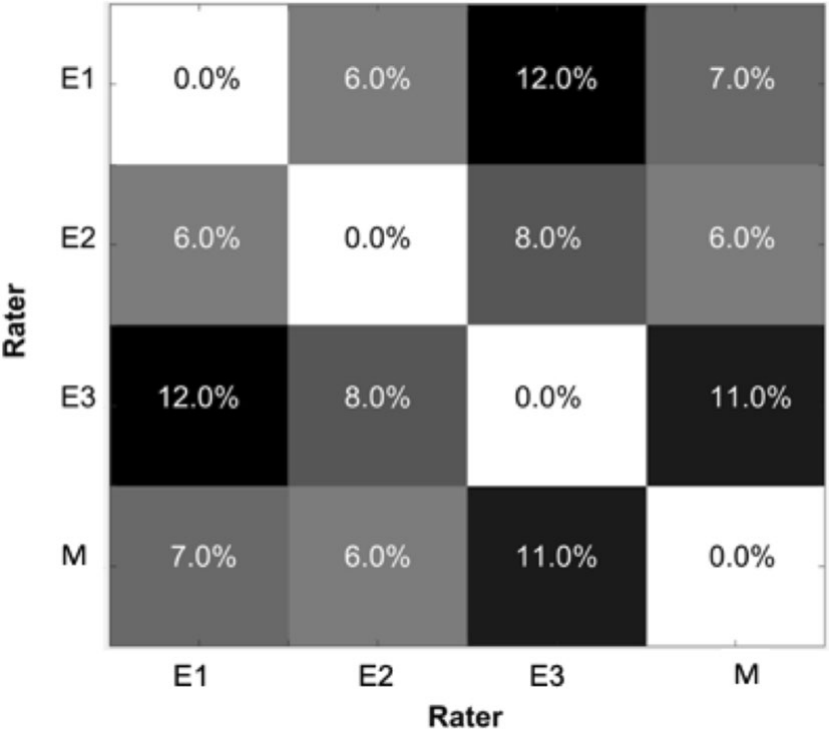
Disagreement percentages between each pair of raters. E1, E2, E3 represents expert 1, 2, and 3, respectively, while M indicates the CNN-DS method. Disagreements rate is displayed in gray-scale blocks; the darker the block, the higher the disagreement rate. Figure 1 was created using Matlab R2017b (https://www.mathworks.com).

Similarly, the linear models obtained for each pair of raters in terms of measurements showed that the CNN-DS method was highly similar to those between the two neuroradiology experts, while rater 3 appeared to be more likely showing dissimilarity (Fig. 1). The average disagreement rate between experts {E1, E2, E3} was 0.09 ± 0.02, whilst the corresponding value between “method-expert” was a comparable 0.08 ± 0.02.

**Figure 2:**
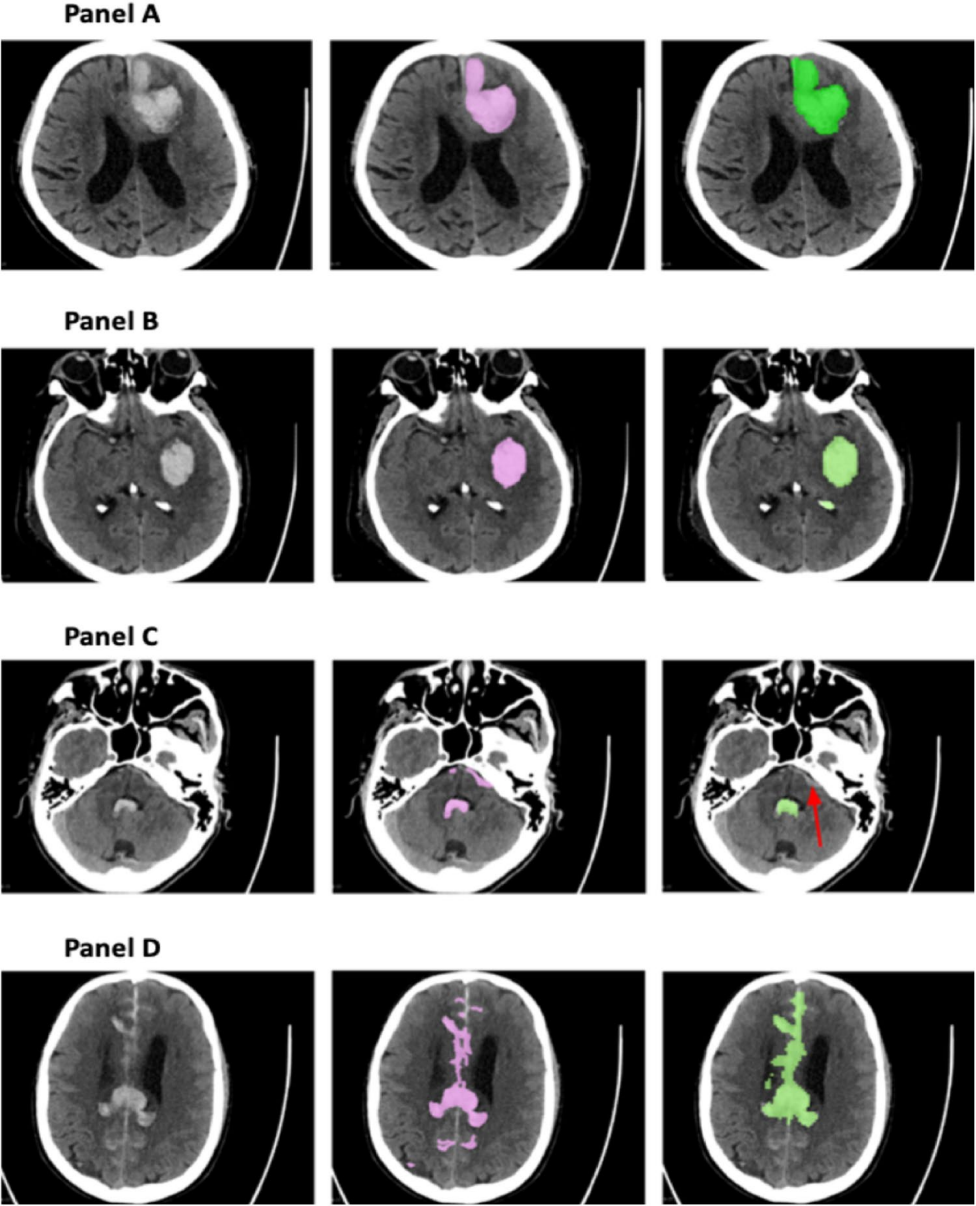
Examples showing the segmentation outcomes using the CNN-DS method. In each panel, the left, middle, and right images are the original CT slice, the ‘ground truth’ labels, and the CNN-DS predicted segmentation, respectively. The pointing arrows indicate the error. (**A**) Represents a case where the CNN-DS method demonstrates an expert-level performance. (**B**) Shows a false positive instance where a calcified structure is labelled as a hemorrhagic area due to its Hounsfield Unit values being higher than those of its surrounding tissues. (**C**) Shows a false negative example in which the CNN-DS method identified part of the hemorrhage but missed some blood close to the bone. (**D**) Illustrates a more complicated case of complex hemorrhage where the discrepancies between the ‘ground truth’ and the predicted segmentation cannot necessarily be attributed to erroneous prediction.

Figure 2 shows several examples of segmentation output of the CNN-DS method compared to radiologist raters; demonstrating high accuracy with a Dice score of 0.88 (Panel A), false positive (Panel B), false negative (Panel C), and more obvious discrepancies (Panel D). As the examples showed, even though it might be perceived as false negative, the areas missed by our CNN-DS method are controversial parts for which even radiologists do not agree on; *e.g.*, as shown by the voxels of missed subarachnoid hemorrhage identification in the sulci of bilateral parietal lobes (Fig. 2D).

### Efficiency evaluation

The training of the CNN-DS model was executed for 500 iterations with a batch of size one (i.e., one image), taking 18 h to complete. Once trained, the neural network could complete the segmentation of any new whole-head CT image of size 128 × 128 × 64 in < 1.0 s. Here, the average execution time was 0.74 ± 0.07 s on a single graphics processing unit (GPU). This included all the pre-processing steps, demonstrating superb efficiency.

### Comparison with previous methods

Table 3 summarizes the result of the experiments comparing the CNN-DS method against the patch-wise U-Net, slice-based U-Net, and the PItcHPERFeCT methods. The bold values in the table indicate the method that achieved the best performance for a particular metric. In all metrics except recall, the CNN-DS method developed in the present study outperformed the others. The apparent higher recall of the PItcHPERFeCT method compared to CNN-DS was associated with (i) a cost of higher processing time; i.e., CNN-DS was 1900 times faster than PItcHPERFeCT (0.74 ± 0.07 s verses 1412.34 ± 20.05 s), and (ii) a large precision hit, i.e., a drop from 0.85 for CNN-DS to 0.63 for PItcHPERFeCT, suggesting the promising potential of CNN-DS with clinical utility in support of fast decision making.

**Table 3:** Segmentation quantitative performance.

Method	Dice score	Precision	Recall	F1 score	Processing time (s)
Patch-wise U-Net^33^	0.74 ± 0.09	0.73 ± 0.17	0.76 ± 0.09	0.74	9.4 ± 0.2
Slice-based U-Net^20^	0.80 ± 0.7	0.78 ± 0.10	0.84 ± 0.08	0.80	12.3 ± 3.6
PItcHPERFeCT^28^	0.76 ± − 0.11	0.63 ± 0.15	**0.98 ± 0.01**	0.77	1412.34 + 20.05
CNN-DS (present study)	**0.84 ± 0.06**	**0.85 ± 0.07**	0.83 ± 0.07	**0.84**	**0.74 ± 0.07**

### Ablation study

**Figure 3:**
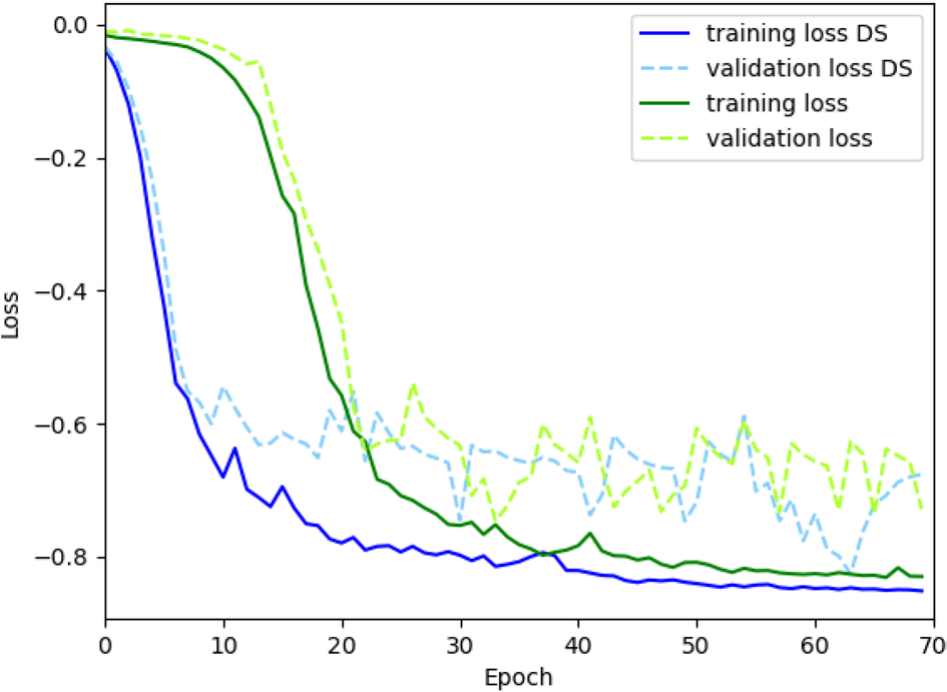
The training and validation loss for the U-Net model with and without deep supervision. The x-axis indicates the number of epochs, which is the number of times the deep learning model has passed through the entire training data during the training phase. The y-axis represents the loss value which implies how well the model behaves after each epoch; the lower the loss, the better a model. The dashed lines show the validation losses while the solid lines show the training losses. For the model with the deep supervision (blue lines), the training loss converges at a considerably faster rate, and the converged loss value is lower than the converged value of the model without deep supervision (green lines).

Figure 3 shows the training and validation for the two models, comparing CNN with and without deep supervision (DS). For the model with the deep supervision, the training loss converges at a considerably faster rate while the converged loss value is lower than the converged value of the model without deep supervision, demonstrating that the model with deep supervision had an improved robustness.

As shown in Table 4, removal of deep supervision introduced a respectively 0.05, 0.04, and 0.06 drop in the dice coefficient score, precision, and recall, respectively. The CNN with DS showed slightly higher processing time, presumably attributed to the skip connections that can induce a delay in the GPU (graphical processing unit) buffer de-allocation as the input of the skip connection must be kept in memory until the end of the skipped block to be added to the output. The bold values in the table indicate the method that achieved the best performance for a particular metric.

**Table 4:** Segmentation quantitative performance for the two models with and without deep supervision (DS).

Method/evaluation	Dice score	Precision	Recall	F1 score	Processing time (s)
Without DS	0.79 ± 0.11	0.81 ± 0.09	0.77 ± 0.09	0.74	**0.72 ± 0.10**
With DS	**0.84 ± 0.06**	**0.85 ± 0.07**	**0.83 ± 0.07**	**0.84**	0.74 ± 0.07

## Discussion

In this study, we developed and evaluated a fully automatic deep-learning solution to accurately and efficiently segment and quantify hemorrhage volume, using the first non-contrast whole-head CT images taken during clinical stroke management. We also compared the performance of this novel U-Net convolutional neural network with deep supervision (CNN-DS) solution with that of the currently available patch-wise U-Net solution^33^, slice-based U-Net^20^, and the random forest based PItcHPERFeCT solution^28^. The results suggested that the CNN-DS is highly accurate at segmenting hematoma, as demonstrated by a Dice similarity coefficient of > 88%. Additionally, agreement analysis examining the automated deep-learning method compared to the human experts revealed that the CNN-DS approach reached an expert-level reliability. Moreover, with an average running time of just 0.7 s, the fully automated solution exhibited its potential in aiding real-world clinical stroke management, where fast segmentation plays a critical role^9,10,15^.

Our data must be interpreted with caution. First, a main general limitation in the field of medical image analysis applying deep learning technologies is the lack of access to sufficiently large datasets^34,35^, and our work is no exception. We were REB-approved to use the very first non-contrast CT images of 200 patients during the approved study time, but had to randomly selected 55 for use in the project due to limitations on expert time with manual input. Future studies with increased sample size will verify and test the generalization of the research finding. Even so, our data were from multiple hospitals, included different kinds and levels of hematoma complexities, and involved a variety of scanner types, making the sample representative of the diverse patient population.

This need of larger datasets is typically compounded by the need for multiple voxel-wise labelling of each image set by multiple clinical experts, making ground-truth reference preparation a time-consuming and tedious task and challenging to perform manually by busy clinicians^36,37^. Despite such obstacles, we succeeded in engaging a team of six qualified raters who independently labelled a dataset of 55 CT images of 512 × 512 ×  [45–66] voxels (i.e., of a total of 57,671,680 voxels) for training and validation. Our testing dataset, though relatively small, consisted of randomly selected new cases that were completely unknown to the model, and thus involved no possibility of data leakage as with other cross validation methods.

Certainly, the raters varied in their experience of evaluating the CT scans and segmenting hematoma, which appeared to be the primary source of the variation in the human–human reliability of the segmentation results (Fig. 1). Despite the difference, the human–human variation was still reasonably low in our study. Here, importantly, even by counting only the highest agreement rate between the two experts with quite similar experience, the CNN-DS based machine–human reliability was only marginally but not statistically lower (0.88 ± 0.05 vs 0.83 ± 0.009). Further research needs to pay a closer attention to improved uniformity of the human rater quality in generating the best possible ground-truth reference standard. This is particularly true with the training dataset, a portion of which was labelled by non-neuroradiology experts. Nevertheless, the supplementary labelling was used only for training the CNN-DS and a high-performance model on testing data was yielded, confirming the superior capacity of the U-Net based solution in effectively dealing with the noise in the training data^38,39^. Future work may leverage recent advances in deep learning from noisy annotations^40^.

Meanwhile, even though the chances do get better with larger sets of correctly labelled data, no dataset can possibly cover absolutely all-different variants of hemorrhagic stroke, whilst the theoretical ‘ground-truth’ reference can always be subject to human errors and thus never be perfectly accurate. To this end, our on-going effort is to explore techniques such as active learning to identify a set of unlabeled images^41^, as well as creating automated simulation datasets with known labels (unpublished data under review), whose ‘ground truth’ would be most beneficial to increase the accuracy of the method in future work.

Even with these limitations, our study has contributed to the development of clinically translatable software tools in support of hemorrhagic stroke management. There are several clinical scenarios where detection and quantification of the volume of hematoma are of unequivocal importance^9,11,17,22,23^. First, in a triage system, such software tools can be used to identify the presence of hemorrhage in patients and alert physicians for immediate attention, reducing the turnaround time for patients with such critical conditions. Also, once a patient is diagnosed with hemorrhagic stroke, a physician can launch the software to obtain information about the volume and location of the hematoma, and consequently develop effective intervention strategy. In addition, segmentation software can be useful in evaluating repeated imaging performed for hematoma growth estimation^42,43^. In this case, the software will be used to provide a numerical value of the hematoma volume change between consecutive CT scans of the same patient performed within a short period. Such knowledge is valuable for prognostication as well as to guide future care planning. In this paper, we have aimed to realize the utility of the CNN-DS method to be established into a potential software tool for aiding all three clinical scenarios.

Our research also contributes to the advancement of computer science. Although several methods have been described in the literature to segment intracranial hemorrhage, they all suffer from some major drawbacks such as failing to balance the trade-off between accuracy and time-efficiency, sensitivity to initialization states, and lack of evaluation on CT images from multiple institutes^9–17^. Even the most recently developed deep learning solutions have often not been able to realize the targeted level of performance^18,19^. As one of the favorable methods standing out in the literature, the PItcHPERFeCT solution achieved a Dice similarity coefficient of 91%^28^. Even so, the level of accuracy is sabotaged by a slow processing speed (i.e., the extremely long running time), due to prolonged pre-processing steps such as skull stripping, and template registration, and computing several hand-engineered features for every voxel^28^. As a result, segmentation of one whole-head CT image can take more than 20 min, which makes it less suitable for solving real-world problems in clinical settings. Similarly, the novel CNN-DS method outperformed the more recently introduced methods for segmenting hematoma volume based on other deep learning models^20,33^, both in accuracy measures and running time.

Future work in this research line will test the possibility to build the CNN-DS method into an “easy-to-operate” software application with a user-friendly interface. This will enable future clinical translation of deep-learning innovations into bedside tools for point-of-care usage.

## Conclusion

In this study, we have developed and evaluated a fully automatic deep-learning method, namely convolutional neural networks with deep supervision (CNN-DS), for segmentation and quantification of hematoma volume in CT images. This method demonstrated human-expert level reliability while being highly time-efficient compared to other established machine-learning approaches. The CNN-DS solution has the potential to be deployed in clinical settings in the future to assist physicians in identifying and evaluating hemorrhage and guiding clinical decision-making, leading to improved clinical outcomes for patients with hemorrhagic stroke.

## Methods

### Dataset

This is a secondary use of existing clinical CT scans. Based on the REB approval, 200 patient CT images were obtained: The first non-contrast whole-head CT scans of patients who were newly identified with a primary diagnosis of acute hemorrhagic stroke between January 1st 2011 and January 1st 2018 across Fraser Health, British Columbia. The samples were retrieved using non-probability sampling based on accessibility at the time of data preparation^44^. Patients with history of hematomas or with hemorrhage due to tissue plasminogen activator administration for treatment of ischemic stroke were excluded. Out of the retrieved data, we randomly selected 55 CT image sets (52.7% male; mean age = 64.1 ± 14.9 years) applying a random data generator. The images were then annotated and pixel-wise labeled for use in the study (i.e., there were 780,894,208 labeled voxels per CT scan). The CT dataset contained images that were acquired using several models of CT scanners, including GE Discovery CT750, GE LightSpeed VCT, and Siemens SOMATOM Definition Flash.

### Image preprocessing

Each CT image contained 43 to 60 2D slices (mean: 53.72 ± 7.16) with a matrix size of 512 × 512. Voxel spacing for x and y varied from 0.42 mm to 0.50 mm, and slice thickness ranged from 2.4 mm to 3 mm. Each CT image was first stripped off the patient identifying information prior to further use. Then, each of the anonymized whole-head CT images was resized to a standard space of 128 × 128 (in plane) × 64 (axial slice) voxels using linear and nearest-neighbor interpolation^45,46^. The average voxel size was (1.8, 1.8, 2.4) mm^3^ with no gap.

The non-contrast CT scans had relatively low contrast due to reasons including low-dosage radiation and noise during image acquisition and reconstruction, which is a known source of artefacts with image processing^47^. Therefore, signal intensity normalization was performed, to increase the contrast in highlighting the hemorrhagic areas as typically applied in CT analyses^47^. This was implemented using python^48^ to adjust the grayscale and the brightness components of the images: Hounsfield Unit values less than 0 were mapped to zero, values higher than 100 were clamped to 100, and the values within the range (0, 100) remained the same.

### Creation of reference standard

To create a “ground truth” reference standard for training the CNN-DS model and for evaluating and comparing the performance, each CT scan was manually segmented slice by slice to generate a binary classification of hematoma vs. normal brain tissue. Each voxel was labelled as either zero or one, representing the non-hemorrhagic and hemorrhagic class respectively. Then the processed CT data were separated into two completely non-overlapping subsets through random selection: the training set (n = 45) and a testing set (n = 10).

Segmentation of the testing set and a portion of the training set (n = 15) was performed by a group of three raters, consisting of two experienced neuroradiologists (co-authors of this article TG and HG, with respectively 12 and 10 years of medical imaging experience); and a neuroscience MSc candidate (BC with three years of research training and additional specific training in hemorrhage evaluation and CT image segmentation), whose work was supervised and double-checked per request by a third neuroradiologist (WS, with 20 years of experience). Each rater segmented each of the voxels independently, blinded from patient information as well as the decision of other raters. The final label of a voxel was selected based on majority voting of the three raters.

This was supplemented with the segmentation of an additional training dataset (n = 40) by an experienced neurologist (co-authors of this article, GM with 20 years of experience) and a computing science PhD candidate (AA with three years of research training and specific training and supervision in hemorrhage evaluation and CT segmentation from GM). Each voxel was labelled with the final decision agreed upon by both raters.

### Overall workflow

A convolutional neural network with deep supervision (CNN-DS) model for automatic segmentation of hematoma was developed using the training set. Then, the model was evaluated using the novel testing set consisting of data previously unseen by the model. Out of the available data, 10 images were selected randomly as the testing test, while the rest of the 45 images were used for training. Based on the segmented voxels, the hemorrhage volume was estimated automatically applying probability threshold, *e.g.*, 0.50.

### Neural network architecture

**Figure 4:**
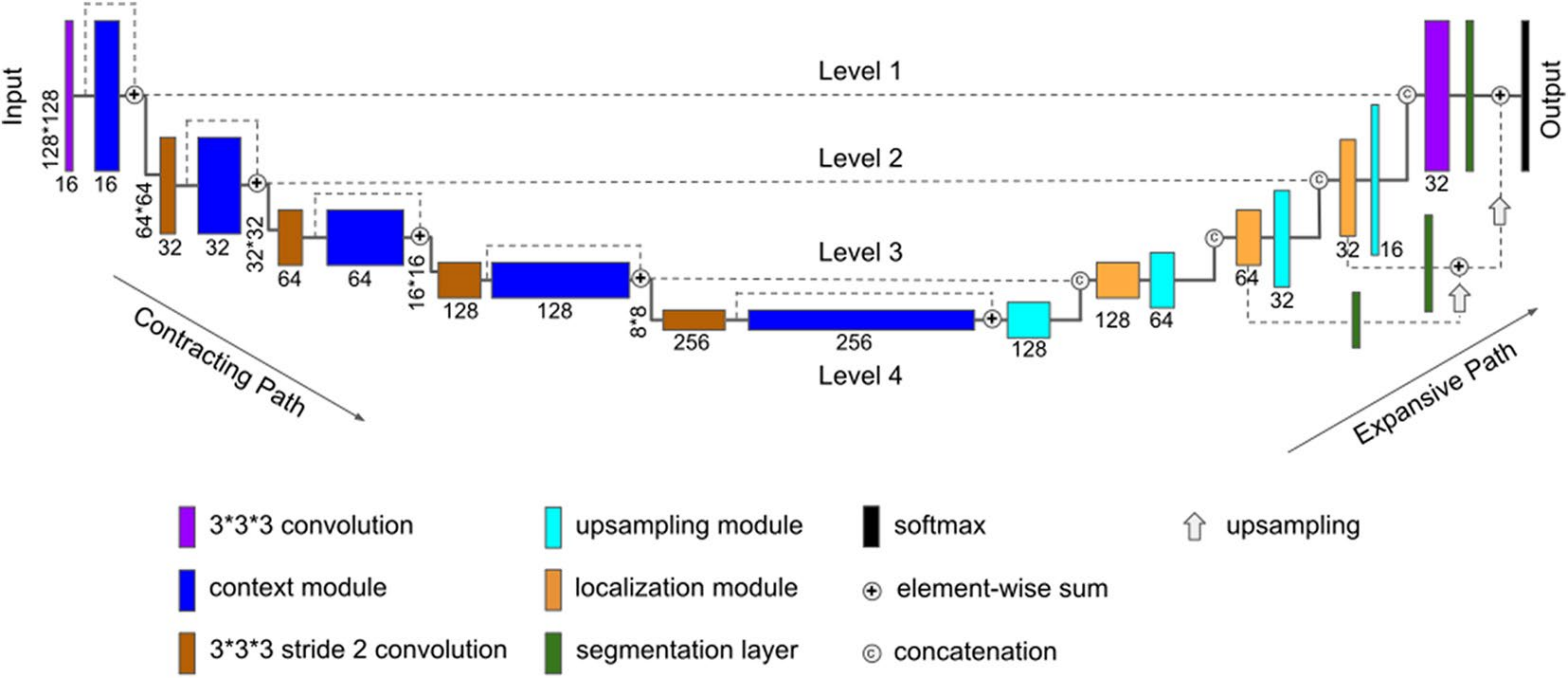
The architecture of the CNN-DS neural network model. The dashed lines show the skip connections while the solid lines show the normal ones. The neural network learns features of the image based on a hierarchy framework starting with simple features such as edges and shapes and going through more complex and high-level features in the deeper levels. The contracting path extracts the features while the expansive path reconstructs the final labelling. Google Slides was used to produce this figure (https://docs.google.com/presentation).

The U-Net architecture is a CNN network designed for training end-to-end for dense segmentation, *i.e.*, producing voxel-wise labelling. The present study was developed based on the improved version of the U-Net architecture^33,49^. The network consists of a contracting path and an expansive path, each with a depth of four layers, as shown in Fig. 4.

The contracting path consisted of image filters to capture context and the convolutional layers followed by rectified linear units (ReLU) and max-pooling layers. Each context module was a residual block^50^ consisting of two 3 × 3 × 3 convolutional layers with a dropout layer in between. The application of context modules was repeated and connected by 3 × 3 × 3 convolutions with stride 2^50^.

The expansive path is applied to perform voxel labelling. It has a similar architecture as the contracting path. However, with the expansive path, the feature map resolution was increased by replacing max-pooling operators with up-sampling operators^50^. Each up-scaling module consisted of a 3 × 3 × 3 up-sampling layer followed by a 3 × 3 × 3 and a 1 × 1 × 1 convolution. The output of the upscaling module was concatenated with corresponding feature maps from the contracting path, and the result was fed to a localization module containing a 3 × 3 × 3 followed by a 1 × 1 × 1 convolution. The expansive path recovers the original spatial size image from feature maps using up-sampling operations. Localization modules, i.e., the two convolutions, are applied in each level to help the model learn to assemble a more precise output.

Deep supervision^51^ was utilized by integrating segmentation layers at different levels of the network and merging them using element-wise summation to produce the final network output. The output of segmentation layers, as shown in Fig. 4, were first upsampled before being fed into the element-wise summation. To produce precise automated segmentation, high-resolution features (image properties that the CNN learns from the training set) from the contracting path were concatenated with the up-sampled feature maps. Feature maps were injected into the deeper layers of the network to mitigate the problem of the vanishing gradients. In each of such down-sampling steps, the number of channels were doubled for the feature map.

The segmentation layers consisted of 3 × 3 × 3 convolution layers. Both input and output of the network are an image of size 128 × 128 × 64 voxels, with one channel representing the Hounsfield Unit values for each voxel and the probability map for the input and the output image, respectively. A threshold of 0.5 was applied on the probability map to yield the final labels.

### Neural network training

The training dataset was further split randomly in two independent subsets: a set of 40 images for training, and a set of 5 images for validation. The parameters of the neural network were updated during the training process using the training set, to minimize the difference between the predicted segmentation masks against the segmentation of the reference standard (commonly referred to as ground truth). Dice similarity coefficient was used for multi-class segmentations as the loss function of the network, as defined by the formula^52^:

$$loss =-\frac{1}{\left|L\right|} \sum \limits_{l\epsilon L}^{.}\frac{2{\sum }_{i}^{\left|g\right|}{g}_{l}^{i}{p}_{l}^{i}}{{\sum }_{i}^{\left|g\right|}{(g}_{l}^{i}{+p}_{l}^{i})}$$

where *g* and *p* are the label vectors for the predicted segmentation and the reference standard, respectively, *L* = {0,1} is the label space with 1 indicating a voxel belonging to the hemorrhage region and 0 otherwise, and $\left|g\right|$=$\left|p\right|$ is the size of vectors *g* and *p*.

A grid-search was used to tune the hyper-parameters of the neural network based on the validation set. We tuned the network depth {3,4,5,6}, learning rate {${10}^{-2},5*{10}^{-3},{10}^{-3},5*{10}^{-4},{10}^{-4}$}, and dropout rate {0.3, 0.4, 0.5}. The Adam optimizer^53^ was used to minimize the loss function. Table 5 shows the final values for the tuned hyper-parameters and the fixed ones. During the training, if the validation loss did not improve for ten consecutive epochs, the learning rate was divided by two. We also incorporated an early stopping mechanism, which stopped the training if no further improvement in validation loss was observed after 50 consecutive epochs.

**Table 5:** The model hyper-parameters.

Hyper-parameter	Value
Network depth	4
Initial learning rate	0.005
Dropout rate	0.3
Batch size	1
First moment estimate	0.9
Second moment estimate	0.999

### Performance evaluation metrics

To assess the ability of the developed CNN-DS method to segment accurately, several approaches were employed. First, the segmentation mask predicted by the CNN-DS method was compared with the reference standard using the Dice similarity coefficient, precision, and recall.

Additionally, to evaluate the reliability of the CNN-DS method, the agreement rate between the neuroradiology experts and the automation method were compared using standard Cohen’s kappa coefficient^54,55^. For n ∈ {1,…,N} and i,j ∈ {1,2,3}, let ${K(Ei,Ej)}_{n},$ be the kappa coefficient computed by annotation of image n by experts Ei and Ej, and let ${K(M,Ei)}_{n}$ be the kappa coefficient computed by annotation of image n by our method and expert Ei, where N is the number of test images. The kappa measures for all the image voxels were grouped into two disjoint sets, where the first set, $\{{K(Ei,Ej)}_{n}\}$, includes all “human–human” comparisons while the second set, {${K(M,Ei)}_{n}\}$, includes “human–machine” comparisons, where “machine” refers to the CNN-DS segmentation method. The size of each set is *R* × *N*, where *R* is the number of raters, and as mentioned above*,* N is the number of test images (in our case, R = 3, N = 10).

If it can be shown that the “human–human” and the “human–machine” comparisons come from the same distribution, it can be concluded that the CNN-DS method performs at a human-expert level, where the “human–machine” agreement rate is indistinguishable from that of “human–human”. This hypothesis was tested using the Mann–Whitney U test at a 0.05 significance level and presented adopting a previously reported procedure. Because there are no meaningful matching between the coefficients from the first group, {K(Ei,Ej)n}, with the second group, {K(M,Eij)n}, we used the appropriate statistical for unpaired data^56^. As three raters each manually segmented the 10 testing images independently, 30 samples were yielded for each comparison, satisfying the recommended sample size for performing the statistical tests^53^.

Also, the predicted volume of the hemorrhage was compared with volumes measured by each neuroradiology expert using the disagreement measure. The hemorrhage volume is computed by multiplying the number of hemorrhage voxels in the binary label image by the corresponding voxel size. For raters *i* and *j*, the disagreement measure was defined as:

$$\frac{1}{N}\sum \limits_{k=1}^{N}\frac{ ({V}_{i,k}-{V}_{j,k})}{(\frac{{V}_{i,k}+{V}_{j,k}}{2})}$$

where ${V}_{i,k}$ and ${V}_{j,k}$ are the volumes measured for image *k* by raters *i* and *j*, respectively, and *N* is the number of images. Moreover, to measure how biased the raters are against each other, we fit a linear model $y = \beta x + \alpha$, where x and y denote volume measurements of the two raters to be evaluated.

### Comparison with other ML methods

The performance of the CNN-DS method was compared to that of three established ML methods in the literature for hematoma segmentation, namely the original patch-wise U-Net^33^, slice-based U-Net^20^, and PItcHPERFeCT^28^. Dice score, precision, recall, F1-score (showing a tradeoff between precision and recall) and processing time of each method was obtained on the testing set (n = 10) and compared with each other using non-parametric statistics.

PItcHPERFeCT is a hemorrhage segmentation tool utilizing random forests algorithm^28^. It was selected for comparison with the CNN-DS method because of the high Dice score that was reported previously for PItcHPERFeCT. This high accuracy is achieved through extraction of extensive hand engineered features along with using prior knowledge such as template of the healthy brain^28^. This makes it an interesting choice as it can be examined whether such hand-engineered feature selection and use of prior knowledge are advantageous compared to CNN-DS method, which fully automatically learns the features. Previous research has noted that several pre-processing steps in PItcHPERFeCT can result in a reduced speed and efficiency, while it may not always be applicable to certain clinically relevant cases with intraventricular hemorrhage and subarachnoid hemorrhages^28^.

The original Patch-wise U-Net method on the other hand is a deep neural network based approach, which follows a U-shaped CNN architecture as shown in Fig. 4. However, instead of being able to directly use the whole image as the input to the network as with the CNN-DS method, with the original patch-wise U-Net method, several patches must be extracted from the images before being fed to the network as input^33^. The original patch-wise U-Net method also does not utilize skip connections, likely affecting the efficiency. This method was chosen due to the characteristics of the clinical dataset. It is well documented that typically, medical image segmentation tasks can suffer from lack of sufficient training samples. Patch-wise U-Net addresses this problem by partitioning the image into several small patches, and augments the data via a variety of transformations on the patches^33^. This operation leads to increased sample sizes for training. However, by feeding the neural network with patches, the classifier loses the context for those patches so that segmentation quality can be negatively impacted. The CNN-DS adopts the original patch-wise U-Net model but overcomes its drawbacks, which will be examined through the comparison.

The backbone of the slice-based U-Net is a classic U-Net^20^. However, instead of the patches, image slices are fed into the network. The U-Net architecture utilizes batch normalization to limit drift of the activation outputs, and 50% dropout to minimize overfitting^20^. Several operations are applied on the images before feeding to the network. First, windowing is performed by applying a threshold of 30 to 130 HU to the original grayscale CT images. Then, images are normalized by subtracting the mean and dividing by the standard deviation of gray levels. Next, the images are denoised by applying curvature driven image denoising^20^. Finally, morphological closing operation was applied on the manually segmented hemorrhage region. As the dataset used for our study incorporated images from three hospitals, instead of an uni-site as with the original work^20^, the present study finding is expected to help determine the algorithm’s generalizability to CT scans from multiple institutions.

### Ablation study

We performed additional experiments to isolate the contribution of the deep supervision mechanism to the CNN algorithm. We trained two CNN networks where one utilized deep supervision in the architecture and the other did not, but were similar otherwise. The backbone architecture is shown in Fig. 4.

### Software and programming information

Codes for the study were developed in Python3 (https://www.python.org/download/releases/3.0/) with use of the open source Keras 2.2.2 library and SimpleITK python library^46^. The experiments were executed on a workstation with one Nvidia Tesla K40 GPU. The software 3D Slicer (version 4.8.1) was used for image processing and labelling of the CT images in creation of ground truth^57,58^. Among the three baseline methods for comparison to ours, we used the public code available for reproducing the PItcHPERFeCT, and re-implemented the other two methods based on the published algorithms.

### Statistical analyses

All statistical analyses were performed using RStudio version 1.1.383 (RStudio, Inc., Boston, MA, https://www.rstudio.com), R 3.2.3 (The R Foundation for Statistical Computing, Vienna, Austria, https://www.r-project.org), Python 2.7.12 (https://www.python.org), and sklearn Version 0.19.2 (https://pypi.org/project/scikit-learn/).

### Ethics

This research protocol has received harmonized Research Ethics Board review approval from Fraser Health Authority and Simon Fraser University (FHREB 2016-113). This research project did not involve the enrolment of any new participants. The project consisted of the de-identification and secondary analyses of existing non-contrast CT data of adult patients. The project was conducted with a waiver of informed consent, as approved by the Fraser Health Authority and the Simon Fraser University Human Research Ethics Boards. All methods carried out in this study were in accordance with the ethical standards of the national research committee, Tri-Council Policy Statement: Ethical Conduct for Research Involving Humans (TCPS), and with the 1964 Helsinki Declaration and its later amendments.
